# Long-COVID Clinical Features and Risk Factors: A Retrospective Analysis of Patients from the STOP-COVID Registry of the PoLoCOV Study

**DOI:** 10.3390/v14081755

**Published:** 2022-08-11

**Authors:** Michał Chudzik, Mateusz Babicki, Joanna Kapusta, Żaneta Kałuzińska-Kołat, Damian Kołat, Piotr Jankowski, Agnieszka Mastalerz-Migas

**Affiliations:** 1 Department of Internal Medicine and Geriatric Cardiology, Medical Centre for Postgraduate Education, 01-813 Warsaw, Poland; 2 Department of Family Medicine, Wroclaw Medical University, 51-141 Wrocław, Poland; 3 Department of Internal Medicine and Cardiac Rehabilitation, Medical University of Lodz, 70-445 Lodz, Poland; 4 Department of Experimental Surgery, Medical University of Lodz, 90-136 Lodz, Poland; 5 Boruta Medical Center, 95-100 Zgierz, Poland

**Keywords:** long-COVID, brain fog, COVID-19, post-COVID-19 syndrome, fatigue

## Abstract

Despite recovering from the acute phase of coronavirus disease (COVID-19), many patients report continuing symptoms that most commonly include fatigue, cough, neurologic problems, hair loss, headache, and musculoskeletal pain, a condition termed long-COVID syndrome. Neither its etiopathogenesis, nor its clinical presentation or risk factors are fully understood. Therefore, the purpose of this study was to retrospectively evaluate the most common symptoms of long-COVID among patients from the STOP COVID registry of the PoLoCOV study, and to search for risk factors for development of the syndrome. The registry includes patients who presented to the medical center for persistent clinical symptoms following severe acute respiratory syndrome coronavirus 2 (SARS-CoV-2) infection. The analysis included data from initial presentation and at three-month follow-up. Of the 2218 patients, 1569 (70.7%) reported having at least one symptom classified as long-COVID syndrome three months after recovery from the initial SARS-CoV-2 infection. The most common symptoms included chronic fatigue (35.6%\), cough (23.0%), and a set of neurological symptoms referred to as brain fog (12.1%). Risk factors for developing long-COVID syndrome included female gender (odds ratio [OR]: 1.48, 95% confidence intervals [CI] [1.19–1.84]), severe COVID-19 (OR: 1.56, CI: 1.00–2.42), dyspnea (OR: 1.31, CI: 1.02–1.69), and chest pain (OR: 1.48, CI: 1.14–1.92). Long-COVID syndrome represents a significant clinical and social problem. The most common clinical manifestations are chronic fatigue, cough, and brain fog. Given the still-limited knowledge of long-COVID syndrome, further research and observation are needed to better understand the mechanisms and risk factors of the disease.

## 1. Introduction

The coronavirus disease (COVID-19) pandemic has been ongoing for over two years, and during this time 531,542,973 cases have been reported worldwide, of which 6,310,705 have been fatal and 502,198,989 have recovered [1]. As it has continued, new reports have emerged regarding both severe acute respiratory syndrome coronavirus 2 (SARS-CoV-2) and the diagnostic and therapeutic recommendations of COVID-19 [2,3]. In addition, as a result of enormous human and financial investment, COVID-19 vaccines were developed in a matter of months and are now the most rapidly invented and deployed formulations [4]. Moreover, in late 2021 and early 2022, the first effective oral antiviral drugs targeting SARS-CoV-2 became available [5,6]. All of this, along with subsequent mutations of the virus, has led to a gradually decreasing mortality rate due to COVID-19 [5,6,7].

Despite this, many patients present with persistent clinical symptoms after the disease has resolved, and this can affect up to 30% of patients [8]. The reported symptoms were very diverse and mainly included: fatigue, hair loss, neurological-cognitive disorders (concentration, memory, and speech disorders), referred to as brain fog, cough or even dyspnea. Patients also complained of chest pains, palpitations, joint pains, headaches, olfactory and taste disorders, and gastrointestinal problems. Psychiatric complications, including anxiety, depression, and even post-traumatic stress disorder have also been recorded. The severity and duration of these symptoms are not uniform. In some patients, symptoms resolve spontaneously, while some patients report persistent symptoms for up to 12 months after acute COVID-19 [9,10,11,12,13].

The literature on persistent symptoms after COVID-19 is still scarce. Moreover, the term used to describe this disease is still not clearly established. The literature contains many terms such as long-COVID, post-COVID-19 syndrome, and post-COVID-19 condition. According to the World Health Organization (WHO) definition, it is a condition in which symptoms persist for at least two months after the disease and cannot be explained by another cause [14]. In contrast, the National Institute for Health and Care Excellence definition of long-COVID includes new or ongoing symptoms 4 to 12 weeks after the acute phase of the illness is diagnosed. Additionally, this definition distinguishes between “ongoing COVID,” which is between 4 and 12 weeks, and “post-COVID-19 syndrome” for symptoms lasting more than 12 weeks [15].

The etiopathogenesis of the disease is also not fully understood. Currently, it is estimated that the cause is complex and consists of persistent inflammation, autoimmunity, and survival of the virus in the body, which may account for the wide variety of symptoms. An important role has also been attributed to the possibility of vascular endothelial damage during infection [16,17].

Interestingly, risk factors have not been clearly established, which potentially include the number of symptoms during COVID-19, old age, female gender, or the presence of chronic conditions such as asthma. However, these factors are not homogeneous for all symptoms classified as long-COVID syndrome. Moreover, most of the available data concern hospitalized patients [11,13,18,19,20]. Taken together, this indicates how much is still unknown and how important it is to delve further into this problem, especially among outpatients.

The aim of this study was to evaluate the most common symptoms of long-COVID among patients of the STOP COVID registry, and to look for risk factors for long-COVID syndrome, including chronic fatigue and brain fog.

## 2. Materials and Methods

### 2.1. Methodology

This was a retrospective study based on analysis of data from 2218 patients obtained from the STOP COVID registry (PoLoCOV (ClinicalTrials.gov identifier—NCT05018052)). The STOP COVID registry includes only patients from Poland. This registry contains medical information of patients presenting to health centers for persistent clinical symptoms after COVID-19 and subsequent follow-up visits at 3 and 12 months. The patient registry includes both inpatients and outpatients from 1 September 2020 to 30 September 2021. Data obtained from visit 0 and up to 3 months were used for the purpose of this review. The program is still ongoing, and future publication of the entire follow-up results, including the visit after 12 months, is planned.

Inclusion criteria were consistent throughout the study and included:

(a) diagnosis of COVID-19 (initially by reverse transcription polymerase chain reaction test only, then also by antigen tests—according to the changes to the rules for COVID-19 diagnosis in Poland);
(b) age ≥ 18 years;
(c) written informed consent to participate in the study.

Exclusions included only:

(a) age < 18 years

or

(b) No consent to participate in the study.

Before participating in the study, patients were informed about the study objectives and methodology, after which they gave informed consent to participate in the study.

At individual visits, patients completed health questionnaires regarding persistent symptoms up to three months after completion of isolation, and were physically examined. At the first visit, patients provided their sociodemographic data, details of chronic conditions, clinical symptoms present during COVID-19, sites of isolation, and duration of clinical symptoms. The most common symptoms of COVID-19 were assessed, including, fever, subfebrile state, chills, cough, headache, myalgia, weakness, dyspnea (subjective), olfactory/taste/hearing disturbances, and chest pain. Subsequently, the patient subjectively rated the feeling of severity of COVID-19 course on a four-point scale. Based on all of the above data, the severity of COVID-19 course was rated on a four-point scale.

The criteria for each point included:

- 0 points (asymptomatic/mild course)
  ○ asymptomatic course
  ○ paroxysmal symptoms lasting no more than 3 days
- 1 point (mild course)
  ○ home isolation
  ○ subjective assessment by the patient of a score of ‘1’ on a scale of 0–3
  ○ duration of symptoms less than 7 days
- 2 points (moderate)
  ○ subjective assessment by the patient of a score of ‘2’ or ‘3’ on a scale of 03
  ○ duration of symptoms lasting between 7 and 14 days
  ○ the presence of dyspnea and fever ≥ 38 °C
- 3 points (severe course)

one of the following criteria: hospitalization with a diagnosis of pneumonia, respiratory failure, intensive care unit (ICU) treatment, respiratory support, or thromboembolic complications during hospitalization/home isolation

or

in the case of home isolation: symptoms persisting for more than 14 days, temperature above 38 °C, dyspnea, oxygen saturation <94% persisting for at least 3 days, or patient’s subjective assessment of “3” on a scale from 0–3 points

Anthropometric measurements, including height and weight, were also taken, and obese patients with a body mass index ≥30 were singled out. Influenza vaccination was also evaluated, and after the introduction of COVID-19 vaccination in Poland, vaccination status during the course of infection was included. A person was considered vaccinated if he or she had completed the baseline schedule, regardless of the type of formulation used.

The following long-COVID symptoms were assessed: cough, dyspnea, chronic fatigue, hair loss, olfactory disturbances, headache, and osteoarticular pain. In addition, the presence of impaired memory and concentration, difficulty in speaking, inability to focus, and lowered mental acuity were assessed, all of which were defined as brain fog [10].

According to the WHO definition, long-COVID was diagnosed with the presence of at least one symptom in a period of 12 weeks from the end of the illness. The condition may include either new symptoms that appeared during COVID-19, or those that persisted and/or worsened during this period [14].

The study was conducted according to the guidelines of the Declaration of Helsinki and approved by the Bioethics Committee of the Wroclaw Medical University, Poland (approval number 232/2022).

### 2.2. Statistical Analysis

The variables analyzed were qualitative, quantitative, and ordinal. Basic descriptive statistics were used to describe the study group and the prevalence of long-COVID.

Logistic regression analysis was used to assess risk factors for the development of long-COVID syndrome. Univariate models were constructed where the dependent variable was the prevalence of long-COVID syndrome and the explanatory variables were, respectively, sociodemographic variables (age, gender), influenza and COVID-19 vaccination status, burden of chronic diseases, and the course of COVID-19 (place of isolation, symptoms during the disease, number and duration of symptoms, and severity of the disease course). Identical univariate models were also constructed for explanatory variables including chronic fatigue and brain fog.

Subsequently, multivariate logistic regression models with the best subset selection were built for the explanatory variables (presence of long-COVID syndrome/brain fog/chronic fatigue) using the Akaike criterion. In these models, the explanatory variables were statistically significant variables obtained by univariate analysis—separately for each explanatory variable.

Results were presented as odds ratio (OR) with 95% confidence intervals (Cl) and p-value, where *p* < 0.05 was considered significant. Analysis was performed using Statistica 13.0 software from StatSoft (TIBCO Software, Palo Alto, CA, USA).

## 3. Results

### 3.1. Characteristics of the Study Group

A total of 2218 patients were included in the analysis, of which 1410 (63.5%) were female, with a mean age of 53.8 ± 13.5 years. At least one chronic condition was recorded in 1569 (70.7%) patients, the most common being hypertension (37.2%), obesity (31.2%), hyperlipidemia (19.8%), and diabetes (10.4%). A detailed description of the study group is shown in Table 1.

### 3.2. Long-COVID Disease Picture and Its Risk Factors

The prevalence of long-COVID-19 syndrome in the study group, detailing the most common clinical manifestations shown in Table 2. In univariate analysis, the risk of developing long-COVID syndrome was shown to be associated with the presence of at least one chronic condition, with the risk increasing with the number of conditions. An analogous relationship was observed for persistent chronic fatigue. 

**Figure 1:**
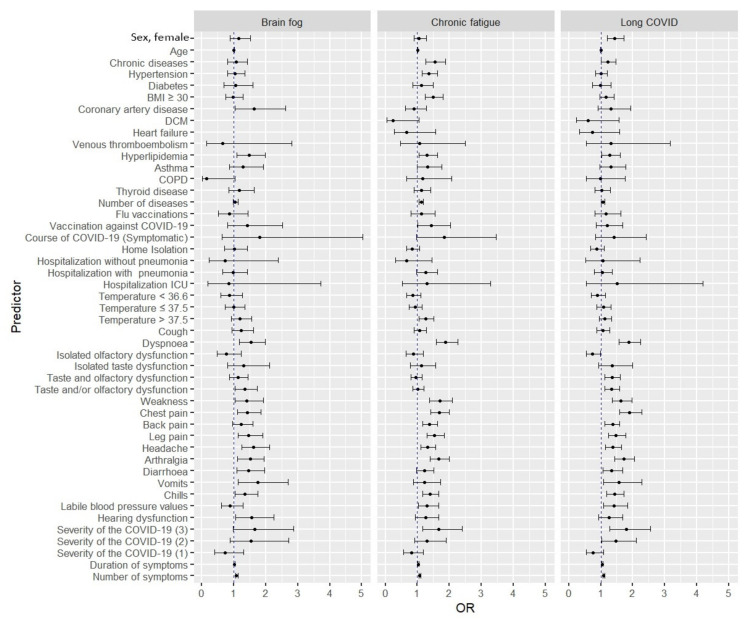
Univariate analysis of risk factors for developing long-COVID syndrome, chronic fatigue, and brain fog.

Additionally, it was shown that as the severity of COVID-19 increased, the risk of developing long-COVID syndrome increased. In symptom-specific analysis, dyspnea, chest pain, and significant weakness were associated with a higher risk of long-COVID syndrome and persistent chronic fatigue. In both cases, these risks increased with respect to both the number of symptoms manifested and their duration. These factors also increased the risk of developing brain fog (Figure 1). A detailed summary is shown in Table 3.

**Figure 2:**
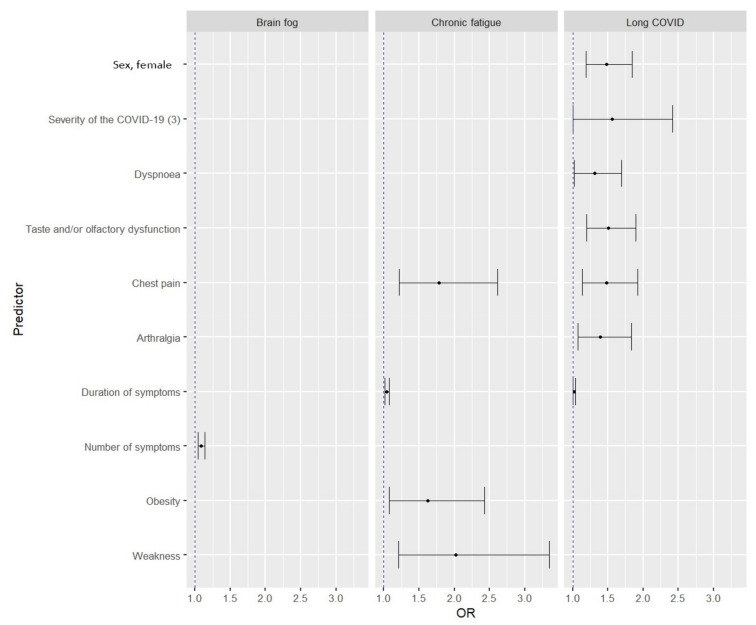
Predictors of the risk of developing long-COVID, brain fog, and chronic fatigue in multivariate models.

Multivariate models showed that female gender and severe COVID-19 predisposed to the development of long-COVID syndrome. Among clinical symptoms, dyspnea, chest pain, and joint pain were associated with a higher risk of long-COVID. For brain fog, only the number of symptoms with which the risk of the disease increased proved to be statistically significant. In contrast, chest pain, significant weakness, and a history of obesity appeared to be risk factors for developing chronic fatigue (Figure 2). A detailed summary of the multivariate analysis is shown in Table 4.

## 4. Discussion

The purpose of this study was to determine the prevalence of long-COVID syndrome and the main symptoms, among patients affiliated with the STOP-COVID registry, that represent the most common clinical manifestations of long-COVID syndrome. Additionally, a search for the risk factors for developing long-COVID syndrome was undertaken. The registry includes both inpatients and post-hospitalization patients. More than half of the post-COVID-19 patients—1440 (65%)—included in the registry reported the persistence of symptoms up to three months after disease resolution. The most common symptoms were chronic fatigue, cough, headache, and a set of neurological symptoms referred to as brain fog.

Our results appear to be consistent with worldwide reports, where these symptoms are the most common manifestations of long-COVID syndrome. In a large analysis conducted among 21,359 patients, as many as 42.3% of them declared the presence of symptoms 30 days after the end of the disease, and 24.1% after 90 days. The main symptoms included problems with concentration, memory, olfactory and/or taste disorders, fatigue, and cough [13]. However, in a study among patients after hospitalization, as many as 75% declared the occurrence of at least one symptom within three months after discharge, of which the most common were dyspnea, cough, fatigue, and difficulty sleeping [20]. In a Canadian study, this percentage was similar, involving 76% of patients [21]. Interestingly, in studies including only patients who had been treated in the ICU, the most common complaints were cough, dyspnea, and fatigue. The authors pointed to persistent respiratory dysfunction after COVID-19 as the reason for this [22].

In our study, hospitalization was not shown to be a risk factor for disease progression. Interestingly, ICU treatment also did not increase the risk of developing long-COVID syndrome, which has also been confirmed in previous studies [23]. In contrast, in both univariate and multivariate analysis, disease severity significantly increased the risk of developing long-COVID, as did female gender, duration of COVID-19 symptoms, and olfactory and/or taste disturbances, arthralgia, and chest pain. Furthermore, univariate analysis indicated that as the number of symptoms in the acute phase of the disease increased, the likelihood of developing long-COVID syndrome increased. Similar observations were made in a database analysis from the “COVID Symptom Study”, where the presence of at least five symptoms increased the “risk” of long-COVID 3.5-fold [9].

Several studies to date have confirmed that females are more likely to develop long-COVID syndrome, similar to chronic fatigue syndrome (ME/CFS). This syndrome is defined as severe fatigue that does not subside after rest, accompanied by malaise, cognitive impairment, pain, and autonomic dysfunction. According to researchers, these conditions are intertwined and mitochondrial dysfunction and metabolic changes are suspected as the cause [18,23,24,25,26]. It should also be considered that females are far more likely to be predisposed to report symptoms, which may also influence the outcome [26].

Another important aspect worth discussing is the effect of vaccination on the risk of long-COVID. However, the impact is unclear, and the data do not provide a clear answer. Nonetheless, early data indicated that the full vaccination schedule reduces the risk of persistent symptoms beyond 28 days, and data from the United Kingdom even indicated a 41.1% reduction in risk [9,27]. Recent data are consistent with our observations and indicate that vaccination is not effective in the prevention of long-COVID syndrome [28]. However, a significant limitation of our observation is the small population of vaccinated individuals and the lack of knowledge about the type of preparation used, so the result should not be generalized. Undoubtedly, further research in this area is necessary.

Fatigue is described as the most common, or second most common, manifestation of long-COVID syndrome [21,22,23]. Its frequency is also variable, with some authors reporting it in up to 80% of patients, with a higher prevalence in hospitalized patients [11,13]. In univariate analysis, we found that elderly patients, those with comorbidities, and those who had severe COVID-19 were significantly more likely to suffer from persistent fatigue, with an influence of both duration and the number of symptoms. Among individual symptoms, our observations confirm previous reports of a direct effect of severe fatigue during COVID-19 on the risk of its prolongation. Chest pain was also associated with an increased risk. One potential cause may be a history of myocarditis, which may have gone undiagnosed during COVID-19 and resulted in decreased physical performance [29].

The characteristic symptom of long-COVID syndrome is brain fog, which includes a set of neurological symptoms that are a consequence of COVID-19. According to the literature, up to one in seven patients may be affected by this condition [30,31]. Despite observations, its mechanisms are still unknown, though one hypothesis is that damage to the nervous system in response to ongoing inflammation alters brain function, particularly in areas related to cognitive function [32,33]. It would seem that univariate analysis, where headache, olfactory and/or taste disorders, and hearing disorders were associated with a higher risk of developing brain fog, could support the present theses. However, in multivariate analysis, only the number of symptoms significantly increased the risk of brain fog. Undoubtedly, this disease symptomatology requires further research and follow-up.

The authors are aware of the limitations of this work, in particular the selection of the study group. The group included patients who had self-referred to the health center due to persistent symptoms after COVID-19 recover and not all patients presented after COVID-19. Therefore, this group is not representative of COVID-19 survivors. Consequently, the results should not be translated to the entire population of COVID-19 survivors. Furthermore, the methodology of the study is also a limitation, as the retrospective nature of data collection carries the risk of memory error, which may affect the reliability of estimating the frequency of individual symptoms. Moreover, collection of information on all complaints, within a specific time frame, may contribute to both underestimation and overestimation of the frequency of individual symptoms. In addition, in estimating risk factors, the authors did not take into account the impact of chronic medications, or medications used during and after infection, which can undoubtedly have a significant impact on the nature and duration of long-COVID syndrome.

On the other hand, the strength of this study is the fact that, to the best of the authors’ knowledge, it is the first such description of patients from Poland. Moreover, it is one of the largest described patient registries with such a high proportion of patients after outpatient COVID-19 treatment, whereas the literature is rich in post-hospitalization observations. This allowed us to assess the impact of the site of isolation on the risk of developing long-COVID as a whole and its individual clinical manifestations. This is very important given that the vast majority of patients with COVID-19 are treated at home.

Despite the limitations of the present study, it represents an important contribution to the knowledge of long-COVID syndrome. Our observations are largely consistent with existing data indicating that long-COVID is a significant problem that affects millions of people worldwide. Indeed, despite ongoing studies and observations, we are still uncertain about its pathogenesis, risk factors, and therapeutic options. Therefore, it should be one of the health priorities in the near future, particularly as some patients report symptoms even 12 months after the disease. Moreover, its clinical course is highly variable, and both our results and worldwide reports show that different risk factors may predispose to particular symptoms, e.g., fatigue, brain fog, or olfactory and/or taste disturbances.

The diversity of risk factors for individual symptoms indicates to us that, for effective prevention and treatment, the syndrome should be considered not only as a whole clinical picture, but also in the context of individual symptoms. These observations may contribute to the identification of risk groups for long-COVID syndrome, fatigue, and brain fog, and may be used to target early diagnostic and clinical interventions to mitigate the sequelae of COVID-19 and enable early recovery. It is important to remember that a prolonged condition leads to absenteeism from work, impaired daily functioning, and thus quality of life. Looking at the number of people who have contracted or will contract COVID-19, this problem poses a tremendous challenge not only to the individual, but also to society and the economy.

## 5. Conclusions

During the three months following the acute phase of COVID-19, persistent clinical symptoms were common. The most common clinical manifestations were chronic fatigue, headache, cough, and brain fog. Severe disease course, duration of COVID-19 symptoms, and female gender, increased the risk of long-COVID syndrome. Obesity is an independent risk factor for persistent chronic fatigue, as is significant fatigue during the acute phase of COVID-19. Given the still-limited knowledge of long-COVID syndrome, further research and observations are needed to better understand the mechanisms and risk factors of the disease.

## Figures and Tables

**Table 1:** Characteristics of the study group.

	Variable	The Whole Group *N* = 2218 (%)	Female	Male	*p*
Sex	Female	1410 (63.5)	---	---	---
	Male	808 (36.5)	---	---	---
Age (M ± SD)		53.8 ± 13.5	53.84 ± 13.3	53.72 ± 13.7	0.923
Weight [kg] (M ± SD)		79.6 ± 17.2	73.3 ± 15.1	90.69 ± 14.9	<0.001
Height [cm] (M ± SD)		169.1 ± 9.1	164.5 ± 6.2	177.1 ± 7.8	<0.001
BMI (M ± SD)		21.3 ± 9.1	20.4 ± 9.1	22.7 ± 8.9	<0.001
Chronic diseases		1569 (70.7)	981 (69.6)	588 (72.7)	0.123
Chronic diseases	Hypertension	826 (37.2)	476 (33.8)	350 (43.3)	<0.001
	BMI > 30	692 (31.2)	388 (28.0)	304 (38.3)	<0.001
	Diabetes	230 (10.4)	125 (8.9)	105 (13.0)	0.002
	Coronary artery disease	134 (6.0)	66 (4.7)	68 (8.4)	0.005
	DCM	17 (0.8)	5 (0.4)	12 (1.5)	0.007
	Heart failure	26 (1.2)	12 (0.9)	14 (1.7)	0.100
	Venous thromboembolism	24 (1.1)	16 (1.1)	8 (1.0)	0.917
	Hyperlipidemia	440 (19.8)	269 (19.1)	171 (21.2)	0.258
	Asthma	217 (9.8)	155 (10.9)	62 (7.7)	0.014
	COPD	51 (2.3)	30 (2.1)	21 (2.6)	0.571
	Thyroid disease	374 (18.9)	328 (23.3)	46 (5.7)	<0.001
Place of Isolation	Home	1887 (85.0)	1229 (86.9)	658 (79.5)	
	Hospital—without pneumonia	33 (1.5)	17 (1.2)	16 (1.9)	0.204
	Hospital—with pneumonia	279 (12.6)	140 (9.9)	139 (17.2)	<0.001
	Hospital—ICU	19 (0.9)	11 (0.8)	8 (1.0)	0.781
Flu vaccinations in the previous season		166 (7.5)	110 (7.8)	56 (6.9)	0.505
Vaccination against COVID-19 (*n* = 697)		494 (70.9)	341 (72.9)	153 (66.81)	0.118

COPD—chronic obstructive pulmonary disease. ICU—intensive care unit M–mean. SD—standard deviation. DCM—dilated cardiomyopathy

**Table 2:** Prevalence of long-COVID-19 syndrome in the study group, detailing the most common clinical manifestations.

Symptom	*N* (%)
Long-COVID	1444 (65.1)
Chronic fatigue	789 (35.6)
Headache	661 (29.8)
Cought	510 (23.0)
Brain fog	268 (12.1)
Dyspnoea	137 (6.2)
Hair loss	104 (4.7)
Olfactory dysfunction	98 (4.4)
Osteoarticular pain	91 (4.1)

**Table 3:** Univariate analysis determining the effect of sociodemographic variables, chronic conditions, influenza and COVID-19 vaccination status, and COVID-19 course on the risk of developing long-COVID syndrome, as well as brain fog symptoms and chronic fatigue.

Variable		Long-COVID			Brain Fog			Chronic Fatigue		
		Percentage (%)	OR (95%Cl)	*p*	Percentage (%)	OR (95%Cl)	*p*	Percentage (%)	OR (95%Cl)	*p*
Sex	Female	961 (66.6)	1.44 (1.20–1.72)	**<0.001**	178 (12.6)	1.15 (0.88–1.51)	0.301	509 (36.1)	1.06 (0.89–1.28)	0.493
	Male	483 (59.8)	Ref.	Ref.	90 (11.1)	Ref.	Ref.	280 (34.7)	Ref.	Ref.
Age		-----	1.00 (0.99–1.01)	0.261	----	1.00 (0.99–1.01)	0.582	----	1.02 (1.00–1.02)	**<0.001**
Chronic diseases	Yes	1043 (66.5)	1.23 (1.01–1.48)	**0.035**	193 (12.3)	1.07 (0.81–1.43)	0.624	603 (38.4)	1.55 (1.27–1.89)	**<0.001**
	No	401 (61.8)	Ref.	Ref.	75 (11.6)	Ref.	Ref.	186 (28.7)	Ref.	Ref.
Hypertension	Yes	538 (61.8)	1.00 (0.84–1.21)	0.982	102 (12.4)	1.04 (0.80–1.35)	0.767	331 (40.1)	1.36 (1.14–1.63)	**<0.001**
	No	906 (65.1)	Ref.	Ref.	166 (11.9)	Ref.	Ref.	458 (32.9)	Ref.	Ref.
Diabetes	Yes	149 (64.8)	0.98 (0.74–1.31)	0.914	29 (12.6)	1.05 (0.69–1.59)	0.796	88 (38.3)	1.13 (0.86–1.50)	0.368
	No	1295 (65.1)	Ref.	Ref.	239 (12.0)	Ref.	Ref.	701 (35.3)	Ref.	Ref.
Obesity (BMI ≥ 30)	Yes	468 (67.6)	1.16 (0.96–1.41)	0.122	82 (11.9)	0.97 (0.74–1.28)	0.856	291 (42.1)	1.49 (1.24–1.80)	**<0.001**
	No	954 (64.2)	Ref.	Ref.	180 (12.1)	Ref.	Ref.	486 (32.7)	Ref.	Ref.
Coronary artery disease	Yes	95 (70.9)	1.32 (0.91–1.94)	0.148	24 (17.9)	1.64 (1.03–2.61)	**0.034**	51 (38.1)	0.89 (0.62–1.28)	0.535
	No	1349 (64.7)	Ref.	Ref.	244 (11.7)	Ref.	Ref.	738 (35.4)	Ref.	Ref.
DCM	Yes	9 (52.9)	0.61 (0.23–1.57)	0.296	0 (0.0)	----	---	2 (11.8)	0.24 (0.05–1.05)	0.058
	No	1435 (65.2)	Ref.	Ref.	268 (12.2)	Ref.	Ref.	787 (35.8)	Ref.	Ref.
Heart failure	Yes	15 (57.7)	0.73 (0.33–1.59)		0 (0.0)	----	---	7 (26.9)	0.66 (0.27–1.58)	0.357
	No	1429 (65.2)	Ref.	Ref.	268 (12.2)	Ref.	Ref.	782 (35.7)	Ref.	Ref.
Venous thromboembolism	Yes	17 (70.8)	1.31 (0.54–3.17)	0.555	2 (8.3)	0.66 (0.15–2.81)	0.574	9 (37.5)	1.08 (0.48–2.50)	0.842
	No	1427 (65.0)	Ref.	Ref.	266 (12.1)	Ref.	Ref.	780 (35.6)	Ref.	Ref.
Hyperlipidemia	Yes	306 (69.6)	1.28 (1.03–1.61)	**0.029**	69 (15.7)	1.48 (1.10–1.98)	**0.010**	179 (40.7)	1.31 (1.06–1.63)	**0.012**
	No	1138 (64.0)	Ref.	Ref.	199 (11.2)	Ref.	Ref.	610 (34.3)	Ref.	Ref.
Asthma	Yes	154 (70.5)	1.31 (0.97–1.79)	0.079	32 (14.8)	1.29 (0.87–1.93)	0.206	90 (41.5)	1.32 (0.99–1.76)	0.056
	No	1291 (64.5)	Ref.	Ref.	236 (11.8)	Ref.	Ref.	699 (34.9)	Ref.	Ref.
COPD	Yes	33 (64.7)	0.98 (0.55–1.76)	0.951	1 (2.0)	0.14 (0.01–1.03)	0.054	20 (39.2)	1.17 (0.66–2.07)	0.582
	No	1411 (65.1)	Ref.	Ref.	267 (12.3)	Ref.	Ref.	769 (35.5)	Ref.	Ref.
Thyroid disease	Yes	245 (65.5)	1.02 (0.81–1.29)	0.857	51 (13.6)	1.18 (0.85–1.64)	0.312	141 (37.7)	1.12 (0.89–1.41)	0.346
	No	1199 (65.0)	Ref.	Ref.	217 (11.8)	Ref.	Ref.	648 (35.1)	Ref.	Ref.
Number of diseases		---	1.06 (1.00–1.12)	**0.049**	----	1.04 (0.97–1.13	0.246	----	1.12 (1.06–1.18)	**<0.001**
Flu vaccinations	Yes	113 (68.1)	1.16 (0.82–1.62)	0.404	18 (10.8)	0.87 (0.52–1.45)	0.610	63 (37.9)	1.12 (0.81–1.55)	0.505
	No	1331 (64.9)	Ref.	Ref.	250 (12.2)	Ref.	Ref.	726 (35.4)	Ref.	Ref.
Vaccination against COVID-19	Yes	326 (66.0)	1.21 (0.86–1.69)	0.268	57 (11.5)	1.42 (0.81–2.52)	0.219	197 (39.9)	1.44 (1.02–2.04)	**0.038**
	No	125 (61.6)	Ref.	Ref.	17 (8.4)	Ref.	Ref.	64 (31.5)	Ref.	Ref.
Course of COVID-19	Symptomatic	1412 (65.1)	1.41 (0.83–2.42)	0.207	264 (12.2)	1.81 (0.64–5.04)	0.257	776 (35.9)	1.85 (0.98–3.46)	0.054
	Asymptomatic	32 (57.1)	Ref.	Ref.	4 (7.1)	Ref.	Ref.	13 (23.2)	Ref.	Ref.
Home Isolation	Yes	1202 (64.6)	0.87 (0.68–1.11)	0.280	225 (12.1)	1.01 (0.71–1.43)	0.963	650 (35.0)	0.84 (0.67–1.07)	0.160
	No	242 (67.6)	Ref.	Ref.	43 (12.0)	Ref.	Ref.	139 (38.8)	Ref.	Ref.
Hospitalization without pneumonia	Yes	22 (66.7)	1.07 (0.52–2.22)	0.849	3 (9.1)	0.72 (0.22–2.39)	0.596	9 (27.3)	0.67 (0.31–1.46)	0.319
	No	1422 (65.1)	Ref.	Ref.	265 (12.1)	Ref.	Ref.	780 (35.7)	Ref.	Ref.
Hospitalization with pneumonia	Yes	184 (66.0)	1.04 (0.80–1.36)	0.751	33 (11.8)	0.97 (0.66–1.43)	0.889	113 (40.5)	1.27 (0.98–1.64)	0.066
	No	1260 (65.0)	Ref.	Ref.	235 (12.1)	Ref.	Ref.	676 (34.9)	Ref.	Ref.
Hospitalization ICU	Yes	14 (73.7)	1.51 (0.54–4.2)	0.434	2 (10.5)	0.85 (0.19–3.72)	0.834	8 (42.12)	1.31 (0.53–3.30)	0.552
	No	1430 (65.0)	Ref.	Ref.	266 (12.1)	Ref.	Ref.	781 (35.5)	Ref.	Ref.
Temperature< 36.6	Yes	198 (63.1)	0.90 (0.70–1.15)	0.418	34 (10.8)	0.87 (0.59–1.27)	0.461	102 (32.5)	0.85 (0.66–1.10)	0.217
	No	1246 (65.4)	Ref.	Ref.	234 (12.4)	Ref.	Ref.	687 (36.1)	Ref.	Ref.
Temperature≤ 37.5	Yes	332 (66.4)	1.08 (0.87–1.32)	0.486	60 (12.0)	0.99 (0.73–1.34)	0.948	171 (34.2)	0.93(0.75–1.14)	0.466
	No	1112 (64.7)	Ref.	Ref.	208 (12.1)	Ref.	Ref.	618 (35.9)	Ref.	Ref.
Temperature> 37.5	Yes	782 (66.4)	1.13 (0.95–1.34)	0.178	153 (13.0)	1.20 (0.93–1.55)	0.164	449 (38.1)	1.27 (1.06–1.51)	**0.007**
	No	662 (63.7)	Ref.	Ref.	115 (11.1)	Ref.	Ref.	340 (32.7)	Ref.	Ref.
Cough	Yes	927 (65.6)	1.06 (0.88–1.27)	0.551	182 (12.9)	1.23 (0.94–1.62)	0.131	512 (36.2)	1.08 (0.90–1.29)	0.407
	No	517 (64.3)	Ref.	Ref.	86 (110.7)	Ref.	Ref.	277 (34.5)	Ref.	Ref.
Dyspnoea	Yes	767 (74.4)	1.87 (1.56–2.24)	**<0.001**	153 (14.5)	1.53 (1.18–1.98)	**0.001**	457 (43.2)	1.89 (1.59–2.26)	**<0.001**
	No	677 (58.4)	Ref.	Ref.	115 (9.9)	Ref.	Ref.	332 (28.7)	Ref.	Ref.
Isolated olfactory dysfunction	Yes	126 (58.6)	0.74 (0.55–0.98)	**0.035**	21 (9.8)	0.77 (0.48–1.23)	0.274	71 (33.0)	0.88 (0.65–1.19)	0.411
	No	1318 (65.8)	Ref.	Ref.	247 (12.3)	Ref.	Ref.	718 (35.9)	Ref.	Ref.
Isolated taste dysfunction	Yes	100 (71.4)	1.36 (0.94–1.99)	0.105	21 (15.0)	1.31 (0.81–2.12)	0.275	53 (37.9)	1.12 (0.78–1.58)	0.559
	No	1344 (64.7)	Ref.	Ref.	247 (11.9)	Ref.	Ref.	736 (35.4)	Ref.	Ref.
Taste and olfactory dysfunction	Yes	684 (68.8)	1.35 (1.13–1.61)	**<0.001**	127 (12.8)	1.13 (0.87–1.45)	0.366	349 (35.1)	0.96 (0.81–1.15)	0.682
	No	760 (62.1)	Ref.	Ref.	141 (11.5)	Ref.	Ref.	440 (36.0)	Ref.	Ref.
Taste and/or olfactory dysfunction	Yes	717 (68.6)	1.34 (1.12–1.59)	**0.001**	144 (13.8)	1.35 (1.04–1.74)	**0.022**	374 (35.8)	1.02 (0.86–1.20)	0.865
	No	727 (62.0)	Ref.	Ref.	124 (10.6)	Ref.	Ref.	415 (35.4)	Ref.	Ref.
Weakness	Yes	1107 (68.2)	1.63 (1.35–1.98)	**<0.001**	211 (13.0)	1.41 (1.03–1.92)	**0.030**	628 (38.7)	1.70 (1.38–2.09)	**<0.001**
	No	337 (56.7)	Ref.	Ref.	57 (9.6)	Ref.	Ref.	161 (27.1)	Ref.	Ref.
Chest pain	Yes	708 (73.1)	1.90 (1.59–2.28)	**<0.001**	138 (14.3)	1.43 (1.11–1.85)	**0.005**	409 (42.3)	1.68 (1.41–2.00)	**<0.001**
	No	736 (58.9)	Ref.	Ref.	130 (10.4)	Ref.	Ref.	380 (30.4)	Ref.	Ref.
Back pain	Yes	795 (68.2)	1.38 (1.13–1.59)	**0.001**	153 (13.1)	1.23 (0.95–1.60)	0.111	455 (39.1)	1.38 (1.16–1.64)	**<0.001**
	No	649 (61.6)	Ref.	Ref.	115 (10.9)	Ref.	Ref.	334 (31.7)	Ref.	Ref.
Leg pain	Yes	670 (70.2)	1.48 (1.24–1.78)	**<0.001**	139 (14.5)	1.47 (1.14–1.90)	**0.003**	394 (41.3)	1.54 (1.30–1.84)	**<0.001**
	No	774 (61.3)	Ref.	Ref.	130 (10.3)	Ref.	Ref.	395 (31.3)	Ref.	Ref.
Headache	Yes	837 (68.3)	1.37 (1.15–1.64)	**<0.001**	175 (14.3)	1.61 (1.24–2.11)	**<0.001**	471 (38.5)	1.33 (1.11–1.58)	**0.002**
	No	607 (61.1)	Ref.	Ref.	93 (9.4)	Ref.	Ref.	318 (32.0)	Ref.	Ref.
Arthralgia	Yes	651 (72.3)	1.72 (1.43–2.06)	**<0.001**	133 (14.8)	1.52 (1.12–1.95)	**0.001**	384 (42.6)	1.67 (1.40–2.00)	**<0.001**
	No	793 (60.2)	Ref.	Ref.	135 (10.3)	Ref.	Ref.	405 (30.8)	Ref.	Ref.
Diarrhoea	Yes	302 (70.2)	1.33 (1.06–1.68)	**0.013**	67 (15.6)	1.46 (1.09–1.96)	**0.014**	169 (39.3)	1.22 (0.98–1.51)	0.072
	No	1142 (63.9)	Ref.	Ref.	201 (11.2)	Ref.	Ref.	620 (34.7)	Ref.	Ref.
Vomits	Yes	111 (74.0)	1.57 (108–2.29)	**0.015**	28 (18.7)	1.75 (1.13–2.69)	**0.011**	60 (40.0)	1.22 (0.87–1.72)	0.241
	No	1333 (64.5)	Ref.	Ref.	240 (11.6)	Ref.	Ref.	729 (35.3)	Ref.	Ref.
Chills	Yes	559 (70.1)	1.43 (1.18–1.72)	**<0.001**	113 (14.2)	1.35 (1.04–1.75)	**0.024**	323 (40.5)	1.40 (1.17–1.67)	**<0.001**
	No	885 (62.3)	Ref.	Ref.	155 (10.9)	Ref.	Ref.	466 (32.8)	Ref.	Ref.
Labile blood pressure values	Yes	234 (71.6)	1.41 (1.09–1.83)	**0.008**	36 (11.0)	0.89 (0.61–1.28)	0.519	134 (41.0)	1.31 (1.03–1.67)	**0.027**
	No	1210 (64.0)	Ref.	Ref.	232 (12.3)	Ref.	Ref.	655 (34.6)	Ref.	Ref.
Hearing dysfunction	Yes	153 (69.6)	1.25 (0.93–1.69)	0.146	37 (16.8)	1.55 (1.06–2.26)	**0.024**	89 (40.5)	1.26 (0.94–1.67)	0.111
	No	1291 (64.6)	Ref.	Ref.	231 (11.6)	Ref.	Ref.	700 (35.0)	Ref.	Ref.
Severity of the COVID-19	3	567 (72.9)	1.80 (1.27–2.55)	**<0.001**	117 (15.1)	1.65 (0.97–2.87)	0.075	333 (42.9)	1.67 (1.17–2.40)	**0.004**
	2	416 (68.8)	1.47 (1.03–2.10)	**0.011**	86 (14.2)	1.54 (0.88–2.71)	0.131	224 (37.0)	1.31 (0.91–1.90)	0.146
	1	348 (53.5)	0.76 (0.54–1.08)	**<0.001**	47 (7.2)	0.72 (0.39–1.31)	0.288	174 (26.7)	0.82 (0.56–1.18)	0.284
	0	99 (60.0)	Ref.	Ref.	16 (9.7)	Ref.	Ref.	51 (30.9)	Ref.	Ref.
Duration of symptoms		----	1.05(1.03–1.07)	**<0.001**	----	1.02 (1.00-1.04)	**0.037**	----	1.03 (1.01–1.05)	**<0.001**
Number of symptoms		----	1.09 (10.7–1.12)	**<0.001**	----	1.08 (1.05–1.13)	**<0.001**	----	1.08 (1.06–1.11)	**<0.001**

OR—odds ratio; CI—confidence interval; Ref.—reference; COPD—chronic obstructive pulmonary disease. ICU—intensive care unit; DCM—dilated cardiomyopathy statistically; significant values are in bold with the significance level set at *p* < 0.05.

**Table 4:** Multivariate analysis based on a model incorporating statistically significant predictors from univariate analysis on the risk of developing long-COVID syndrome, brain fog, and chronic fatigue.

Variable		Long-COVID		Brain Fog		Chronic Fatigue	
		OR (95%Cl)	*p*	OR (95%Cl)	*p*	OR (95%Cl)	*p*
Sex, Female		1.48 (1.19–1.84)	<0.001	---	---	---	---
Severity of the COVID-19	3	1.56 (1.00–2.42)	0.045	---	---	---	---
Dyspnoea		1.31 (1.02–1.69)	0.035	---	---	---	---
Taste and/or olfactory dysfunction		1.51 (1.20–1.90)	<0.001	---	---	---	---
Chest pain		1.48 (1.14–1.92)	0.003	---	---	1.78 (1.22–2.61)	0.002
Arthralgia		1.39 (1.07–1.83)	0.015	---	---	---	---
Duration of symptoms		1.02 (1.00–1.04)	0.049	---	---	1.04 (1.01–1.08)	0.015
Number of symptoms		---	---	1.09 (1.04–1.14)	<0.001	---	---
Obesity		---	---	---	---	1.62 (1.08–2.43)	0.019
Weakness		---	---	---	---	2.02 (1.21–3.35)	0.007

## Data Availability

The data presented in this study are available on request from the corresponding author.
